# How specific is second language-learning ability? A twin study exploring the contributions of first language achievement and intelligence to second language achievement

**DOI:** 10.1038/tp.2015.128

**Published:** 2015-09-22

**Authors:** K Rimfeld, P S Dale, R Plomin

**Affiliations:** 1 King's College London, MRC Social, Genetic and Developmental Psychiatry Centre, Institute of Psychiatry, Psychology and Neuroscience, London, UK; 2 Department of Speech and Hearing Sciences, University of New Mexico, Albuquerque, NM, USA

## Abstract

Learning a second language is crucially important in an increasingly global society, yet surprisingly little is known about why individuals differ so substantially in second language (SL) achievement. We used the twin design to assess the nature, nurture and mediators of individual differences in SL achievement. For 6263 twin pairs, we analyzed scores from age 16 UK-wide standardized tests, the General Certificate of Secondary Education (GCSE). We estimated genetic and environmental influences on the variance of SL for specific languages, the links between SL and English and the extent to which the links between SL and English are explained by intelligence. All SL measures showed substantial heritability, although heritability was nonsignificantly lower for German (36%) than the other languages (53–62%). Multivariate genetic analyses indicated that a third of genetic influence in SL is shared with intelligence, a third with English independent of intelligence and a further third is unique to SL.

## Introduction

Learning a second language (SL) is increasingly important in modern global societies; however, surprisingly little is known about the origins of individual differences in foreign language acquisition. Given the importance of SL ability in the modern world, it is striking that only a handful of published studies have used genetically sensitive methods to investigate the etiology of individual differences in SL achievement. To our knowledge, the twin design has been applied in only three studies. A Dutch study using over 1600 12 to 26-year-old twin pairs, reported a high heritability estimate (71%).^1^ However, this study used self-reported aptitude, not measured performance in SL learning. An Australian study with a relatively small sample of 251 adolescent twin pairs investigated teacher-rated achievement in SL learning and reported high heritability estimates (72%) with shared environmental factors explaining 20% of the variance.^2^ The only adequately powered study using non-self-report SL measures was conducted with a subsample of the present study: teacher-rated achievement for 14-year-old twins from the Twins Early Development Study (TEDS) yielded a substantial heritability estimate of 42%, shared environmental influences of 32% and non-shared environmental influences of 26%.^3^ Importantly, this study also showed shared etiology between age 12 achievement in English and SL at age 14, demonstrating substantial phenotypic (0.44) and genetic correlations (0.49) between the first and SL achievement scores. However, these results were based on teacher ratings, and, as twins often have the same teacher for a given foreign language, this measure could lead to rater bias and to an inflated estimate of shared environment. In summary, the few available studies suggest that there is substantial heritability in SL achievement; however, the results to date are mixed, as would be expected, given the diverse measures used in these studies.

It is possible that SL achievement reflects a broader language skill. Indeed, early first language skills have been shown to be closely related to achievement in SL even after a 10-year gap.^4, 5^ We have shown that achievement in the first language (English) is highly heritable in the early school years^6^ and at the end of compulsory education.^7^ In our previous report on SL, we showed that SL at the age of 14 was substantially correlated phenotypically (0.44) and genetically (0.49) with first language achievement scores.^3^

The strongest predictor of SL achievement is a construct called *second language learning aptitude*, which is generally considered as a specific ability for SL learning.^8^ One way to look at this construct is in terms of ability to learn several languages; however, few students take more than one foreign language General Certificate of Secondary Education (GCSEs) and those that do are likely to be self-selected for SL-learning aptitude. SL-learning aptitude is typically measured using language-learning exercises, such as the Modern Language Aptitude Test^9^ that are very similar to the actual learning outcome they are used to predict, although the underlying psychological mechanisms remain poorly understood.^10^ Language-learning aptitude has been hypothesized to include memory, phonetic coding ability, language analytic ability and grammatical sensitivity,^11, 12, 13^ all of which appear to be related to intelligence. For example, both language analytic ability and memory are usually considered important components of intelligence.^10^ Furthermore, it is not clear whether aptitude is something different from intelligence.^14^ We did not have a measure that specifically addresses SL-learning aptitude. However, in addition to investigating whether SL achievement reflects broader language aptitude that includes first language, we were able to address, for the first time, the extent to which SL achievement is even more general in the sense of general intelligence. Intelligence has been shown to be a significant predictor of SL achievement, as well as academic achievement in general.^15, 16, 17, 18^ In terms of genetics, intelligence, such as academic achievement, is highly heritable (~0.50).^19^ For these reasons, it is important to include intelligence in a multivariate genetic investigation of SL achievement.

In summary, the current study goes beyond our previous report in three ways. First, our sample is three times larger. This increased power enabled us to investigate the main SLs studied at school separately, and also allowed for more powerful multivariate genetic analyses. Second, instead of teacher ratings, our analyses were based on standardized examinations (GCSEs) taken at the end of compulsory education in the United Kingdom. Third, we included intelligence in multivariate analyses. These measures allowed us to investigate the extent to which SL achievement reflects a broader language skill (first language achievement) and an even broader cognitive ability (intelligence). We report results for twins with GCSE scores at the age of 16 in English and SL and for whom intelligence scores were also available. We show, for the first time, the results of trivariate analyses investigating the association between intelligence, English and SL achievement.

## Materials and methods

### Sample

The sampling frame for the present study was the TEDS sample. TEDS is a large longitudinal sample involving over 16 000 twin pairs born in England and Wales during 1994–1996. Although there has been some attrition, more than 10 000 twin pairs have remained actively involved in the study. Since infancy, rich cognitive and behavioral data have been collected from the twins, including academic achievement.^20^ The sample is a representative sample of the UK population when compared with data from the National Statistics Office.^6^

The present study included 12 526 individuals (6263 twin pairs) from whom GCSE scores were obtained for English or SL; intelligence scores were available for 4481 individuals (2240 pairs). The sample size for each measure is shown in the results. Children who had major medical or psychiatric problems were excluded from the analyses. Because the present study investigated achievement in first and second languages, children who did not have English as their first language were also excluded from the analyses; however, no information about the extent of bilingualism was available. Zygosity was assessed using a parent questionnaire of physical similarity, which is 95% accurate when compared with DNA testing.^21^ DNA testing was conducted when zygosity was not clear from physical similarity criteria. Both same-sex twin pairs and opposite-sex twin pairs were included in the study, with the overall sample including 2229 monozygotic (MZ) pairs, 2050 same-sex dizygotic (DZ) twin pairs and 1984 opposite-sex DZ twin pairs.

### Measures

We used the GCSE grades for language achievement measures at the age of 16. GCSEs are standardized examinations taken in the United Kingdom at the end of compulsory education. The GCSE courses usually begin at the age of 14 and children choose from a variety of subjects, from traditional academic subjects such as English and mathematics, to history, geography, music and foreign languages. English, mathematics and science are compulsory subjects; all other courses are chosen from a variety of available subjects. Many schools also require students to take at least one modern foreign language course. These foreign language GCSE courses include reading, writing, listening and speaking the SL; however, only one mean exam grade is awarded for each SL GCSE examination. The examinations are graded between A* and G, which we coded from 11 (A*) to 4 (G). Students typically choose 10 or more GCSEs; receiving five or more grades between A* and C (inclusive) is a requirement for further education. All GCSE scores were collected by questionnaires sent by mail or by telephone from the parents or the twins themselves. Parent- and self-reported grades for English were compared with the grades obtained from the National Pupil database for 7367 twins (NPD; https://www.gov.uk/government/uploads/system/uploads/attachment_data/file/251184/SFR40_2013_FINALv2.pdf), yielding a correlation of 0.98, which indicates high accuracy of parent- and self-reported examination scores; data were not available to make this comparison specifically for SL.

The present study used all foreign language GCSE grades available for each student to create a composite, mean SL GCSE score (2765 twin pairs). The most popular foreign languages taken at GCSE level were French (1323 twin pairs), Spanish (407 twin pairs) and German (450 twin pairs). We analyzed these three language grades separately in addition to the mean SL GCSE grade. GCSE English achievement was used as a measure of first language achievement and was computed as the mean of English language and English literature grades.

Intelligence, or general cognitive ability (‘g'), was assessed from Mill Hill Vocabulary score^22^ and Raven's Progressive Matrices.^23^ Mill Hill vocabulary is a test of verbal ability, which consists of multiple-choice items. For each item a single word is presented at the top of the screen. Participants choose an answer that has the closest meaning to the target word. Raven's Progressive Matrices is a non-verbal ability task, consisting of a series of incomplete patterns (‘matrices'). In each case, the participant is asked to identify the missing part of the pattern. These measures were obtained from the twins at the age of 16 using web-based testing. Intelligence, general cognitive ability (‘g'), was indexed as the mean of the standardized verbal and non-verbal scores. Intelligence scores were available for 4481 individuals, as these data were only collected from a subsample of the TEDS twins (two out of four birth cohorts, and therefore a random subsample of participants).

Before genetic analyses, all measures were corrected for age and sex differences using regression, creating standardized residual scores. This procedure is regularly used in TEDS for analyses of twin data to avoid inflation of estimates of shared environment as members of a twin pair are otherwise identical for age and MZ twins are also identical for sex.^24^ For all analyses, outliers beyond three s.d.'s from the mean were removed. Finally, all measures were transformed to the standard normal distribution using the rank-based van der Waerden transformation^25, 26^ to correct for the negative skew. This negative skew, demonstrating a ceiling effect, was similar to that observed in the UK population as illustrated in UK national statistics (NPD; https://www.gov.uk/government/uploads/system/uploads/attachment_data/file/251184/SFR40_2013_FINALv2.pdf).

### Analyses

#### Descriptive statistics across sex and zygosity

The measures were described in terms of means and variance, comparing boys and girls and identical (MZ) and fraternal (DZ) twins; the mean differences for age and sex and their interaction were tested using analysis of variance (ANOVA). We have previously reported full sex-limitation genetic modeling for GCSE achievement and found little evidence for sex differences in genetic and environmental estimates.^7^ We conducted similar analyses specifically for SL achievement in the present study and confirmed our previous findings suggesting significant quantitative, but no qualitative sex differences. Boys have slightly higher estimates for heritability, whereas girls have slightly higher estimates for shared environment. These differences were, however, small and had overlapping confidence intervals. For these reasons, and to increase power in the present study and to decrease the complexity of reporting, all analyses were conducted on the basis of the full sample, combining sexes and including opposite-sex pairs.

#### Phenotypic correlations

Phenotypic correlations were calculated between the composite GCSE SL and GCSE English, between the main GCSE SL languages of French, German and Spanish, and between SL measures and intelligence. The correlations between GCSEs in individual languages were based on a restricted sample and range, as only a minority of students took two or more GCSEs in a SL.

#### Twin method

The twin method was used to estimate the relative contribution of additive genetic (A), shared environmental (C) and non-shared environmental influences (E) for the variance of SL, English and intelligence measures and for the covariance between them. The twin method offers a powerful natural experiment by comparing the similarity of MZ twins to DZ twins, as MZ twins share 100% of their segregating genes, and DZ twins, just as any other siblings, share 50% of their segregating genes.^27^ By comparing twin correlations for MZ and DZ twins, the relative contributions of A, C and E can be estimated. Both MZ and DZ twin pairs growing up in the same family share the same environmental influences; therefore, the correlation between twin pairs for shared environmental influences is assumed to be 1.0. Non-shared environmental influences are assumed to be unique to individuals, that is, uncorrelated between twins and not contributing to similarities between them.

Cross-twin correlations can be used to estimate ACE parameters. A is approximately double the difference between MZ and DZ correlations; C can be calculated by deducting the heritability estimate from the MZ correlations; and E can be calculated by deducting the MZ correlations from unity. E also includes measurement error.^28^ These A, C and E estimates can be calculated more accurately and with confidence intervals using structural equation models with maximum likelihood estimation. We used the structural equation modeling program OpenMx.^29^ Univariate parameter estimates are reported for all measures.

Bivariate genetic analysis extends univariate analysis of variance to the covariance between two variables. Similar to univariate decomposition of variance, the phenotypic covariance between traits can be decomposed into A, C and E components on the basis of cross-twin cross-trait correlations, examining the covariance between twin pairs across different traits (See Supplementary Figure S1). Genetic correlation (*r*_G_) is an index of pleiotropy: it estimates the extent to which the same genes influence two traits independent of the heritability of the traits. By weighting the genetic correlation by the heritabilities of two traits, genetic mediation of the phenotypic correlation can be estimated. An algebraically equivalent representation of the same analysis is the Cholesky decomposition (Supplementary Figure S1b), which is conceptually similar to hierarchical regression. Cholesky decomposition focuses on the extent to which the heritability of one trait is explained by genetic influences on the other trait (path a_12_ in Supplementary Figure S1b). These analyses also decompose covariance into common shared environmental influences (*r*_C_) and non-shared environmental influences (r_E_). Two bivariate genetic analyses were conducted to assess the links between achievement in SL and achievement in first language (English), and assess the links between achievement in SL and intelligence.

Trivariate genetic analysis extends bivariate genetic analysis to consider all three variables simultaneously: intelligence, English and SL. Trivariate genetic Cholesky analysis was used to estimate (1) the extent to which the heritability of SL can be explained by genetic influence that is shared with intelligence and English, (2) how much is explained by English independent of intelligence and (3) how much genetic influence is specific to SL, independent of both intelligence and English.

## Results

Means and s.d.'s are presented in Table 1 by sex and zygosity for five groups: MZ males, DZ males, MZ females, DZ females and DZ opposite-sex pairs. ANOVA results show that the sex, zygosity and their interaction explain only ~1% of the variance on average.

For subsequent analyses, scores were age and sex regressed and normalized using the van der Waerden transformation, as explained in the Materials and Methods section.

### Univariate model fitting

**Figure 1:**
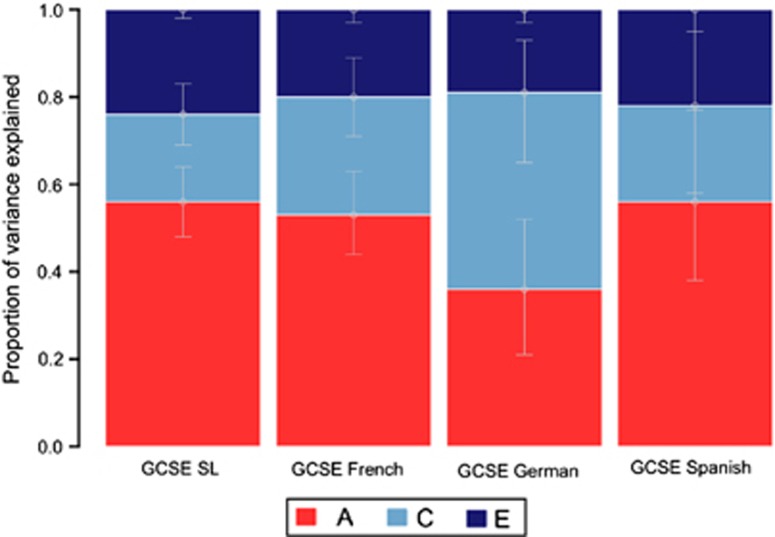
Univariate model-fitting results representing A, additive genetic; C, shared environmental; E, non-shared environmental components of variance for General Certificate of Secondary Education (GCSE) language measures (95% confidence interval (CI)).

Figure 1 shows univariate ACE (additive genetic, shared environmental and non-shared environmental components of variance) estimates for the mean SL score, as well as for French, German and Spanish. SL learning at the end of compulsory education is highly heritable (56% for composite GCSE SL grade). Heritability estimates for French and Spanish are substantial, 53% and 56%, respectively. Shared environmental influence accounted for approximately a quarter (27 and 22%) of the variance. Non-shared environmental influences (*E*) that do not contribute to similarities between the twins accounted for the remaining fifth of the variance (22 and 20%). Interestingly, German language achievement at the age of 16 yields a lower heritability estimate of 36% and a higher shared environmental influence of 45%, although these estimates are not significantly different from French or Spanish. All twin correlations and detailed model-fitting results, together with confidence intervals, are presented in Supplementary Table S1.

### Correlations between SL, English and intelligence

Phenotypic correlations among the three variables are substantial. English and SL correlate 0.70 (0.69–0.72: 95% confidence intervals). Intelligence correlates moderately with both English (0.52; 0.50–0.54) and SL (0.48; 0.45–0.51). Correlations between specific languages are also substantial (0.69–0.79), as shown in Supplementary Table S2. However, the sample size for these correlations was small and possibly not representative as it was limited to students who took more than one foreign language GCSE.

### Bivariate model fitting

**Figure 2:**
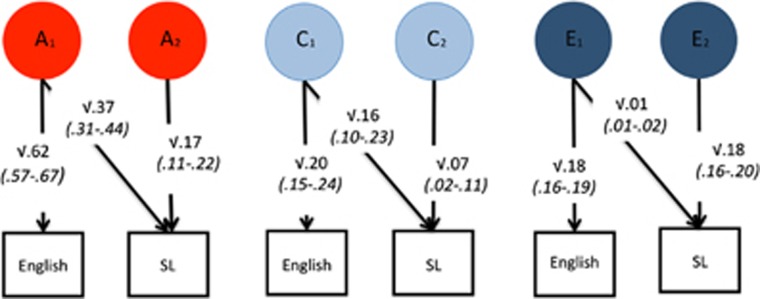
Bivariate model-fitting results for Cholesky decomposition for General Certificate of Secondary Education (GCSE) English and GCSE second language (SL) with 95% confidence intervals (in parentheses).

Figure 2 illustrates the results of bivariate genetic analyses between English and SL. The heritability of SL achievement is 54%, the sum of the two paths √0.37 and √0.17, which differs only slightly from the estimate of 56% from univariate model fitting (Supplementary Table S1). The a_12_ path (see Supplementary Figure S1) of √0.37 indicates that English accounts for 68% (0.37/0.54) of the heritability of SL at the age of 16.

Bivariate genetic analyses conducted between intelligence and SL indicate that intelligence explains 27% (0.15/0.55) of the heritability of SL achievement (see Supplementary Figure S2).

We conducted similar analyses for the specific languages of French, Spanish and German. Supplementary Figure S3 summarizes the results of these analyses. Similar to the results shown for the SL composite in Figure 2, bivariate Cholesky analyses of English as compared with the three languages showed that English accounted for ~80% of heritability of each of the languages (see Supplementary Figure S4). Similar to the results shown in Supplementary Figure S4, bivariate Cholesky analyses showed that intelligence accounted for ~30% of the heritability of each of the languages (see Supplementary Figure S5). In summary, the bivariate results shown in Figure 2 for the SL composite were similar to those that emerged for each of the languages separately; there were some differences in the magnitude of heritability explained by English, but these differences were not statistically significant. It is important to remember that we had much less power to conduct the bivariate analyses using three languages separately as compared with SL composite, as evident from the wide confidence intervals.

### Trivariate model fitting

**Figure 3:**
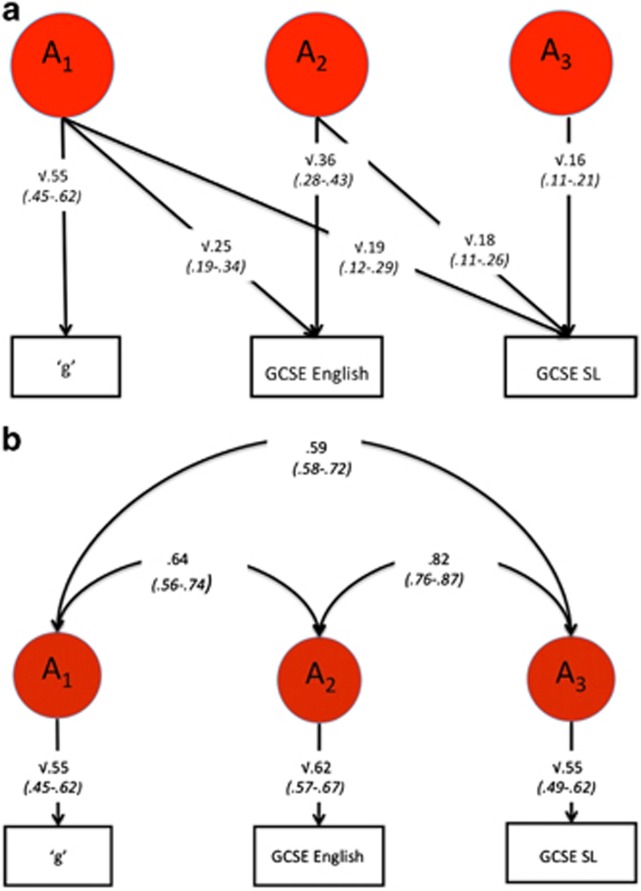
Trivariate genetic Cholesky analyses. (**a**) Trivariate genetic model-fitting results for Cholesky decomposition for ‘g', GCSE English and GCSE SL with 95% confidence intervals (in parentheses); (**b**) correlated factor solution with 95% confidence intervals (in parentheses). GCSE, General Certificate of Secondary Education; SL, second language.

To investigate further the relationships between SL English and intelligence, a trivariate genetic analysis was conducted. Figure 3 presents the genetic results of (a) the Cholesky solution and (b) the correlated factor solution. The Cholesky analysis indicates that 36% (0.19/0.53) of the variance in the heritability of SL can be attributed to intelligence and English, a further 34% (0.18/0.53) of the heritability of SL can be attributed to English independent of intelligence, and 30% (0.16/0.53) of the heritability of SL is unique genetic variance, that is, independent of English and intelligence. Full Cholesky decomposition is shown in Supplementary Figure S6. The correlated factor solution (Figure 3b) yields a genetic correlation of 0.82 between SL and English, suggesting that the same genes largely contribute to these two measures. The genetic correlation between SL and intelligence is 0.59, which is significantly lower than the genetic correlation between SL and English, as seen by their nonoverlapping confidence intervals. Full correlation matrixes, together with confidence intervals, are included in Supplementary Table S3.

## Discussion

We found that most individual differences in SL achievement are accounted for by genetic differences, rather than school, family and other environmental influences. This conclusion holds for both Spanish and French, although there may be less genetic influence and more shared environmental influence for German.

These heritability estimates are higher than those in our earlier study,^3^ which might be because different measures were used. In the present study we used standardized examination scores at the end of compulsory education, as compared with teacher ratings of academic achievement in our earlier report. Secondly, the teacher-rated measure used previously was collected at the age of 14, which is typically in the middle of SL learning. Our current measure was obtained at the end of formal SL education, when individual differences may have become more stabilized.

Our bivariate results demonstrate a general genetic factor of language achievement at the end of compulsory education in the United Kingdom in the sense that achievement in English and SL is influenced to a large extent by the same genes. Furthermore, genetic influence on SL achievement cannot be explained by intelligence alone. SL heritability is just as much explained by English achievement as it is by intelligence, and the genetic bivariate relationship between SL and English is stronger than the bivariate genetic relationship between SL and intelligence. A more comprehensive picture is provided by our trivariate results, which show that genetic influences on intelligence contribute about one-third of the heritability of SL achievement. A further third of the heritability of SL can be accounted for by genetic influence on English independent of intelligence, pointing to a general factor of language. The final third of the heritability of SL is unique to SL, that is, independent of both intelligence and English.

We believe our study is the first adequately powered study to employ standardized examination results for SL learning at the end of compulsory education in order to estimate genetic and environmental influences on the variance and covariance of first and SL achievement and intelligence. There are, however, at least four limitations that need to be acknowledged. First, the usual assumptions about twin method were made, which are described in detail elsewhere.^27^ Second, the instructed language learning studied here could differ from learning in a natural setting, and therefore the results of this study cannot be generalized to SL acquisition outside of classroom settings,^30, 31^ and only apply to those who have chosen to take GCSE in SL. Third, some schools in the United Kingdom require students to take at least one foreign language GCSE, whereas others do not allow pupils to choose more than one; therefore, we could not investigate the genetic and environmental origins of individual differences in choosing one or more foreign language GCSE courses. Furthermore, because SL GCSE is compulsory in some schools but not in other schools, it might not be a random group of students who took one or more foreign language GCSE courses. Finally, the foreign language GCSE examination consists of four parts: reading, writing, listening and speaking, which make it a reliable measure of overall academic achievement in language learning. However, only one composite grade per language is awarded at GCSE level, so that we could not distinguish these different aspects of language learning as they relate to English achievement or intelligence. We created the composite of English language and English literature because there is substantial overlap with the course content measuring reading, writing, speaking and listening skills. Nonetheless, we checked whether analyzing the English language grade by itself yields similar results; the results are highly similar to those shown for the composite measure. It is also noteworthy that both GCSE English and GCSE SL are assessed by standardized examinations, whereas intelligence is not. Thus, it is possible that shared method variance contributes to the correlation between English and SL.

The present results suggest several questions for further research on academic achievement in SLs. Our future research involves longitudinal investigations into SL achievement, for example, a longitudinal analysis exploring how early English achievement and intelligence relate etiologically to SL at the age of 16. We will also explore whether the conclusions presented here for the entire sample hold at the extremes of exceptionally high or low SL achievement. If appropriate samples can be found, multivariate genetic analyses should be conducted in different foreign languages to investigate the extent to which the same genetic and environmental factors influence learning diverse foreign languages. This was not possible in the present study because few students took more than one foreign language GCSE. Furthermore, all of the students in this study were native speakers of English. It would be of considerable theoretical interest to explore the role of first and SL typological distance as an influence on SL etiology, that is, how the differences between languages on various aspects of linguistic structure influence the rate of language learning and achievement. A large body of literature has shown that SL-learning aptitude, learning styles and quality of instruction are significant predictors of the rate of SL learning.^12, 32, 33, 34^ Further research is needed to study the etiology of the associations between these predictors and achievement in SL using a multivariate genetic design, and this is one of our goals for future research. Another goal is to understand the role of specific cognitive abilities, not just general intelligence, on SL achievement. One strategy that could prove useful in this regard is to study individuals with discrepancies between GCSE grades in English and SL.

We have demonstrated here that genes explain a larger proportion of differences between children in SL achievement than do shared environmental influences of school and home environment. It is important to note that genes not only influence the aptitude and achievement of children directly, but also their appetite for knowledge and hence indirectly their eventual achievement. This is an example of genotype–environment correlation; as children grow older they tend to select, modify and tailor their environment on the basis of their genetic propensities.^35^ Genotype–environment correlation may be increasingly important during adolescent development; achievement in language learning could be influenced by how much students use the language outside the school, their interest in the different cultures and self-efficacy.

Achievement at the end of compulsory education is of major, and increasing, importance to society and to individuals because these results are used to make decisions regarding further education and occupation. The findings of our study will become even more important once specific genes responsible for academic achievement in SL learning are identified, unique environmental factors are ascertained and gene–environment interplay is better understood.

## Figures and Tables

**Table 1:** Descriptive statistics

	N	*Whole sample*	*Male*	*Female*	*MZm*	*DZm*	*MZf*	*DZf*	*DZos*	*Sex*	*Zyg*	*Sex × Zyg*	R^*2*^
GCSE English	12 099	8.91 (1.21)	8.69 (1.26)	9.12 (1.14)	8.65 (1.27)	8.75 (1.20)	9.06 (1.13)	9.12 (1.15)	8.92 (1.23)	38.57**	3.09	0.01	0.01
GCSE SL	6896	8.82 (1.42)	8.62 (1.50)	8.96 (1.34)	8.56 (1.51)	8.70 (1.51)	8.91 (1.34)	8.98 (1.33)	8.83 (1.42)	43.45**	3.01	0.8	0.01
Intelligence	4481	0.00 (0.99)	0.05 (1.01)	−0.03 (0.98)	0.00 (0.98)	0.07 (1.05)	−0.08 (0.98)	−0.05 (1.00)	0.06 (0.99)	6.1*	6.48*	0.01	<0.01

Abbreviations: ANOVA, analysis of variance; DZ, dizygotic; f, female; GCSE, General Certificate of Secondary Education; m, male; MZ, monozygotic; *N*, sample size after exclusions (individuals); os, opposite sex; SL, second language; Zyg, zygosity.

Mean (s.d.'s) for GCSE English, GCSE SL grade, and intelligence.

*Note*: The maximum GCSE grade is 11 and the minimum grade is 4, representing grades A* to G. ANOVAs were conducted by selecting randomly one twin per pair testing the main effect of sex and zygosity, and the interaction between them. Results=F statistics, *R*^2^= proportion of variance explained; *P<0.05; **P<0.01.

## Supplementary Material

Supplementary Informations
