# Supplementary material for: How specific is second language-learning ability? A twin study exploring the contributions of first language achievement and intelligence to second language achievement
Source: Transl Psychiatry. 2015 Sep 22;5(9):e638–. doi: 10.1038/tp.2015.128 (PMC5068806; doi:10.1038/tp.2015.128)
Supplement: Supplementary Informations [file tp2015128x1.docx]

**Supplementary Material**

**Tables**

Table S1. Twin intraclass correlations and model fitting estimates for univariate analyses for GCSE language achievement.

Table S2. Phenotypic correlations between GCSE English and intelligence, and the main second languages taken.

Table S3. Correlated factor solution for the trivariate genetic analyses for intelligence, GCSE English and GCSE SL.

**Figures**

Figure S1. Bivariate model of additive genetic (A), shared environmental (C) and non-shared environmental (E) contributions to the correlations between traits. Two algebraically equivalent representations of the bivariate model are shown: (a) correlated factor solution of genetic correlation (*r*_G_), shared environmental correlation *(r*_C_) and non-shared environmental correlation (*r*_E_) and (b) Cholesky decomposition.

Figure S2*.* Bivariate model-fitting results for Cholesky decomposition for ‘g’ (intelligence) and GCSE SL with 95% confidence intervals (in parentheses).

Figure S3. Bivariate estimates for intelligence and GCSE second language achievement measures.

Figure S4. Bivariate model-fitting results for Cholesky decomposition for GCSE English and GCSE French, Spanish and German with 95% confidence intervals (in parentheses).

Figure S5. Bivariate model-fitting results for Cholesky decomposition for intelligence and GCSE French, Spanish and German with 95% confidence intervals (in parentheses).

Figure S6. Trivariate model-fitting results for Cholesky decomposition for intelligence (‘g’), GCSE English and GCSE SL.

*Table S1.*

Twin intraclass correlations and univariate model fitting estimates for additive genetic (A), shared environmental (C) and non-shared environmental (E) components of variance for GCSE language achievement, 95% confidence intervals (in parentheses); N= number of complete twin pairs, MZ=monozygotic, DZ=dizygotic.

|  | **N** | **Intraclass correlations** | |  |  |  |
| --- | --- | --- | --- | --- | --- | --- |
|  |  | **Mz** | **Dz** | **A** | **C** | **E** |
| GCSE SL | 2765 | 0.75 | 0.47 | 0.56 (0.48-0.64) | 0.20 (0.13-0.27) | 0.24 (0.22-0.26) |
| GCSE French | 1323 | 0.81 | 0.54 | 0.53 (0.44-0.63) | 0.27(0.18-0.36) | 0.20 (0.17-0.21) |
| GCSE German | 450 | 0.79 | 0.61 | 0.36 (0.21-0.52) | 0.45 (0.29-0.57) | 0.19 (0.16-0.24) |
| GCSE Spanish | 407 | 0.79 | 0.50 | 0.56 (0.38-0.77) | 0.22 (0.02-0.39) | 0.22 (0.17-0.27) |
| GCSE English | 5911 | 0.81 | 0.51 | 0.62 (0.58-0.67) | 0.20 (0.15-0.24) | 0.18 (0.17-0.19) |

*Table S2.*

Phenotypic correlations between GCSE English and the main second languages. Correlations calculated on one randomly selected twin per pair. N=number of participants; ** p<.01.

|  |  | GCSE English | GCSE French | GCSE German | GCSE Spanish |
| --- | --- | --- | --- | --- | --- |
| GCSE English |  | 1 |  |  |  |
|  | N | 6030 |  |  |  |
| GCSE French |  | 0.69** | 1 |  |  |
|  | N | 2112 | 2113 |  |  |
| GCSE German |  | 0.66** | 0.77** | 1 |  |
|  | N | 872 | 157 | 874 |  |
| GCSE Spanish |  | 0.69** | 0.75** | 0.79** | 1 |
|  | N | 828 | 207 | 50 | 829 |

*Table S3*.

Correlated factor solution for the trivariate genetic analyses, demonstrating the phenotypic correlation (*r*_Ph_), genetic correlation (*r*_G_), shared-environmental (*r*_C_),non-shared environmental (*r*_E_) correlations, and phenotypic correlations (r_ph)_ between intelligence, GCSE English and GCSE SL, 95% confidence intervals (in parentheses).

| ***r*_G_** |  |  |  |
| --- | --- | --- | --- |
|  | Intelligence | English | SL |
| Intelligence | 1.00 |  |  |
| English | 0.64 (0.56-0.74) | 1.00 |  |
| SL | 0.59 (0.59-0.72) | 0.82 (0.76-0.87) | 1.00 |
|  |  |  |  |
| ***r*_C_** |  |  |  |
|  | Intelligence | English | SL |
| Intelligence | 1.00 |  |  |
| English | 0.92 (0.46-1.0) | 1.00 |  |
| SL | 0.99 (0.46-1.0) | 0.84 (0.71-0.96) | 1.00 |
|  |  |  |  |
| ***r*_E_** |  |  |  |
|  | Intelligence | English | SL |
| Intelligence | 1.00 |  |  |
| English | 0.19 (0.12-0.26) | 1.00 |  |
| SL | 0.14 (0.05-0.23) | 0.22 (0.16-0.28) | 1.00 |
|  |  |  |  |
| ***r*_Ph_** |  |  |  |
|  | Intelligence | English | SL |
| Intelligence | 1.00 |  |  |
| English | 0.52 (0.50-.054) | 1.00 |  |
| SL | 0.48 (0.45-0.51) | 0.70 (0.69-0.72) | 1.00 |


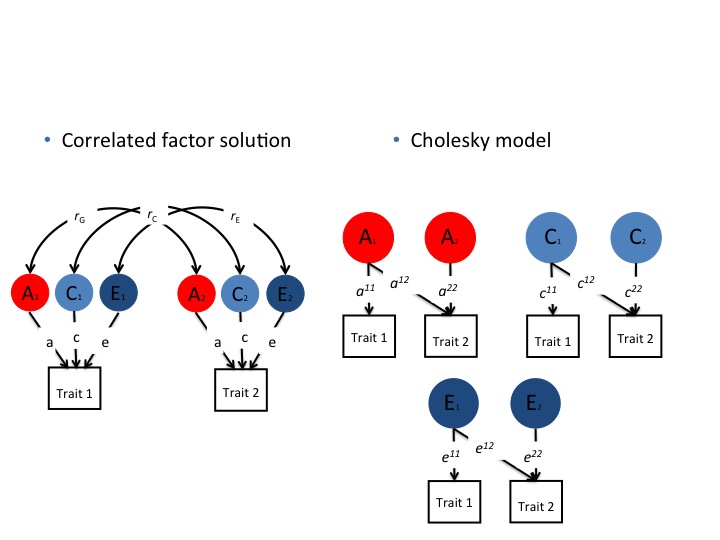


*Figure S1.*

Bivariate model of additive genetic (A), shared environmental (C) and non-shared environmental (E) contributions to the correlations between traits. Two algebraically equivalent representations of the bivariate model are shown: (a) correlated factor solution of genetic correlation (*r*_G_), shared environmental correlation *(r*_C_) and non-shared environmental correlation (*r*_E_) and (b) Cholesky decomposition.

*Figure S2.*

Bivariate model-fitting results for Cholesky decomposition for ‘g’ (intelligence) and GCSE SL with 95% confidence intervals (in parentheses).

*Figure S3.*

Bivariate estimates for additive genetic (A), shared environmental (C) and non-shared environmental contributions to the phenotypic correlations between intelligence and GCSE second language achievement measures. Total length of the bar indicates the magnitude of the phenotypic correlation.


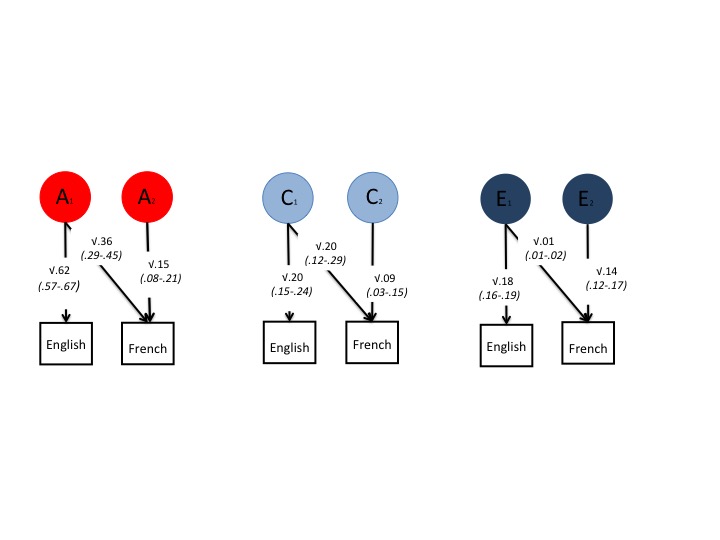


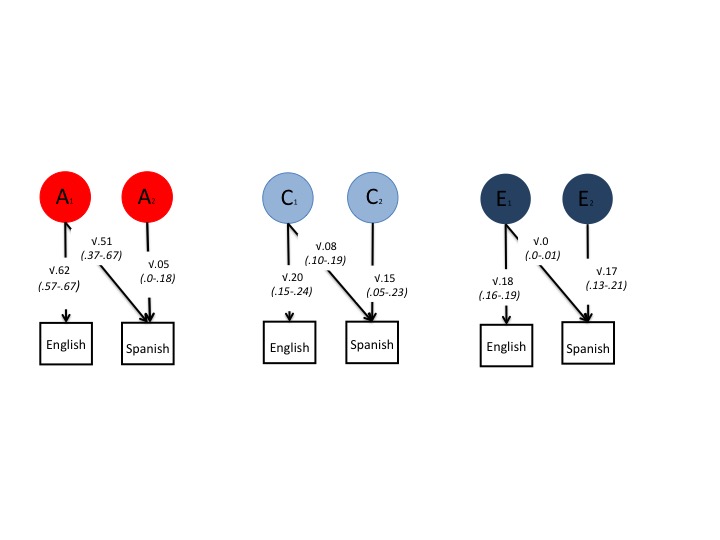


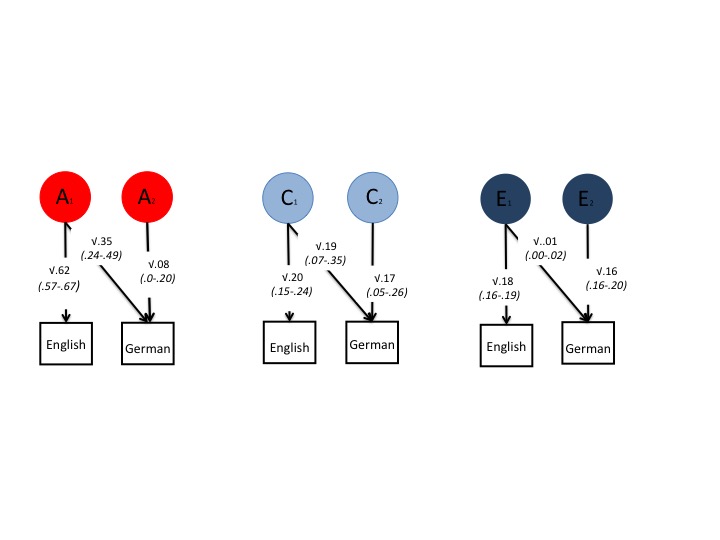


*Figure S4.*

Bivariate model-fitting results for Cholesky decomposition for GCSE English and GCSE French, Spanish and German with 95% confidence intervals (in parentheses).


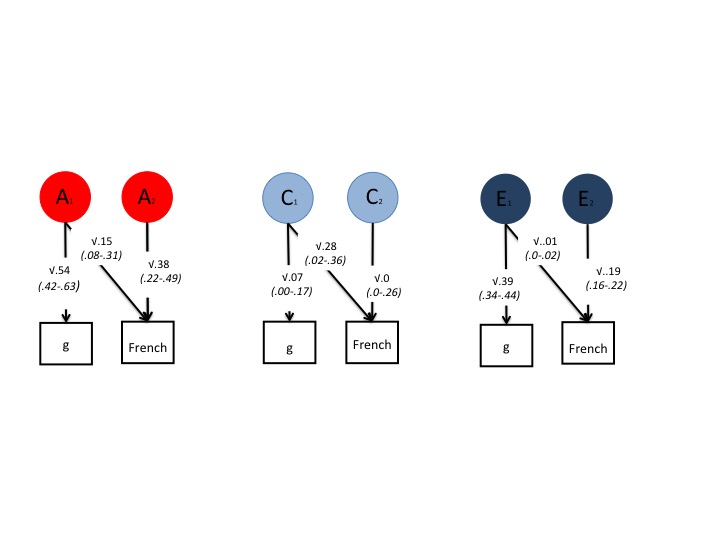


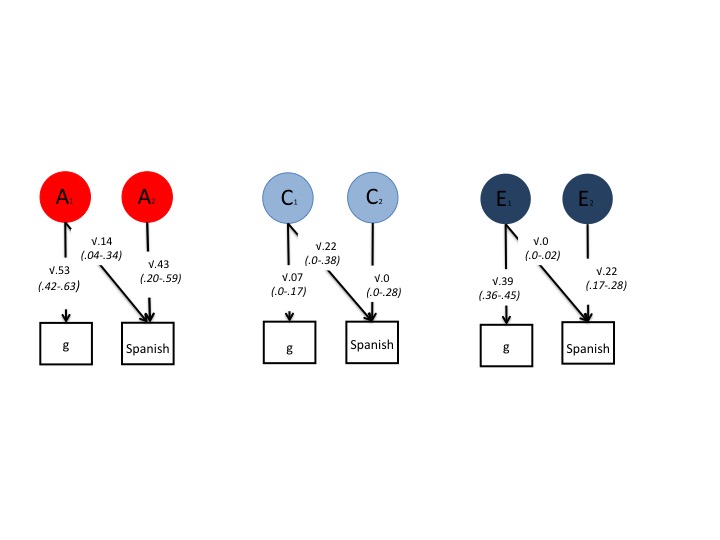


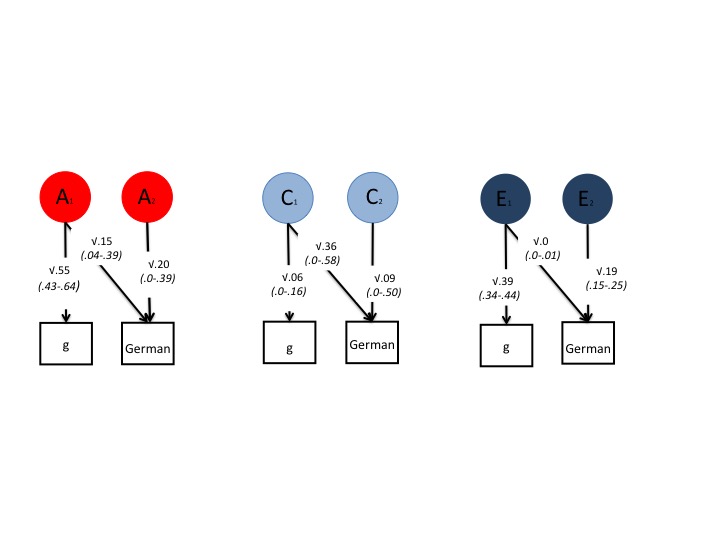


*Figure S5***.**

Bivariate model-fitting results for Cholesky decomposition for intelligence and GCSE French, Spanish and German with 95% confidence intervals (in parentheses).


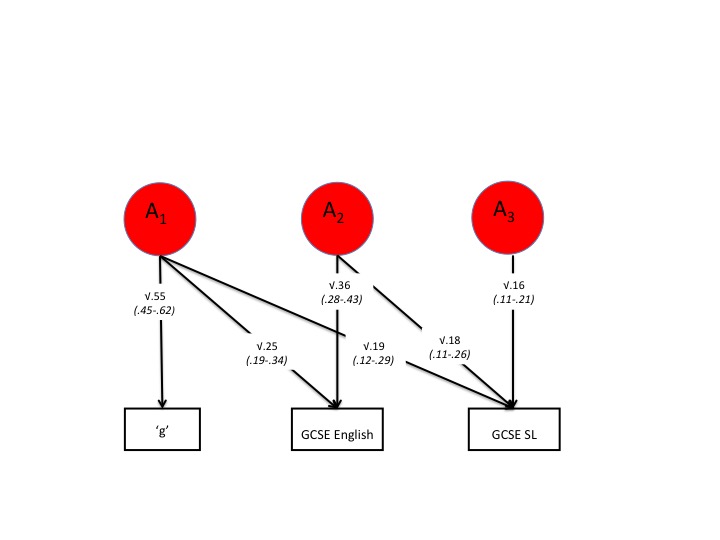

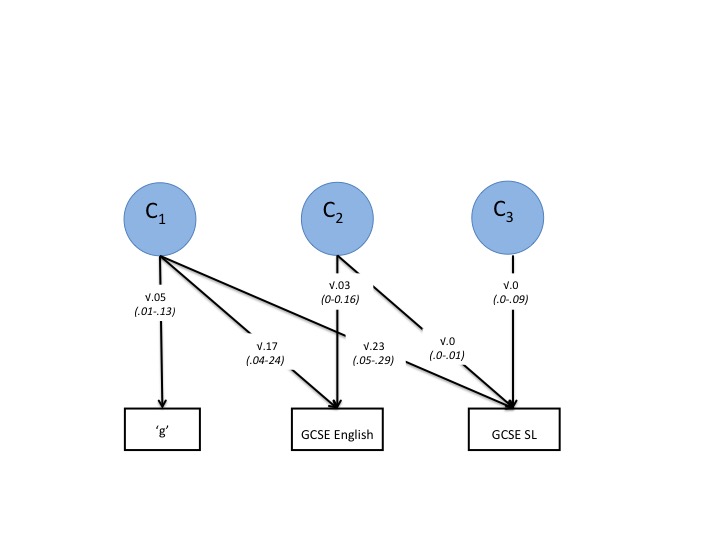


**
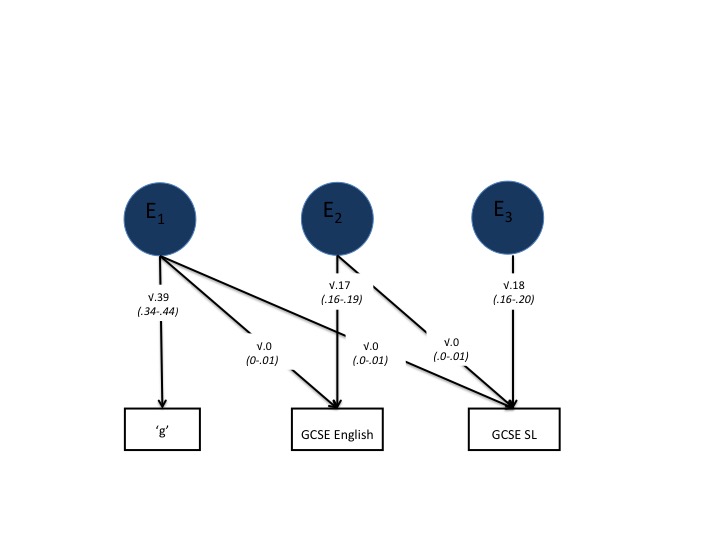
**

*Figure S6.*

Trivariate model-fitting results for Cholesky decomposition for intelligence (‘g’), GCSE English and GCSE SL with 95% confidence intervals (in parentheses); A= additive genetic variance, C= shared environmental variance. E= non-shared environmental variance.
